# Healthcare-associated malaria: a systematic review, 1997 to 2023

**DOI:** 10.2807/1560-7917.ES.2025.30.11.2400393

**Published:** 2025-03-20

**Authors:** Céline M Gossner, Luisa K Hallmaier-Wacker, Harold Noel, Beatriz Fernández Martínez, Danai Pervanidou, Maria Tseroni, Julia Enkelmann, Daniela Boccolini, Diamantis Plachouras

**Affiliations:** 1 European Centre for Disease Prevention and Control (ECDC), Stockholm, Sweden; 2 Santé publique France, Saint Maurice, France; 3 Instituto de Salud Carlos III, CIBERESP, Madrid, Spain; 4 Hellenic National Public Health Organization, Athens, Greece; 5 National and Kapodistrian University of Athens, Athens, Greece; 6 Robert Koch Institute (RKI), Berlin, Germany; 7 Istituto Superiore di Sanità (ISS), Rome, Italy

**Keywords:** Systematic review, Hospital-acquired, healthcare, nosocomial, malaria, protozoa, Plasmodium, Infection Prevention and Control

## Abstract

**Background:**

Malaria is primarily transmitted through mosquito bites; occasionally, direct transmission through blood has been reported. Healthcare-associated infections refer to infections acquired in a hospital or another healthcare setting.

**Aim:**

This systematic review aims to explore determinants of healthcare-associated malaria (HAM) cases.

**Method:**

This review follows the PRISMA guidelines and was registered in PROSPERO (CRD42022309701). We searched five databases for publications on HAM cases published between 1 January 2000 and 7 December 2023. We initiated a data call for HAM cases to public health authorities from 37 European countries. We performed a backward and forward search, reviewed health authorities’ websites, performed searches on Google and the European Scientific Conference on Applied Infectious Disease Epidemiology (ESCAIDE) conference abstracts book.

**Results:**

We identified 37 studies on HAM comprising 55 HAM cases, of which 35 (64%) were infected in Europe, primarily in Spain (nine cases), France and Italy (seven cases each). All cases were infected with *Plasmodium falciparum* except one individual. Fifty HAM cases were hospital inpatients and five were healthcare workers. Five patients died. Flushing of vascular catheters with contaminated heparin/saline solution and manipulation of intravenous catheters were the most frequently reported procedures leading to infection among patients.

**Conclusions:**

While rare, HAM transmission can be fatal. Healthcare-associated malaria is preventable through strict adherence to infection prevention and control procedures. Despite extensive investigations, the procedure leading to infection often remained unknown, highlighting the complexity of investigations. Guidance and protocols for conducting investigations may improve the success rate of such inquiries.

## Introduction

Malaria is a protozoan infection that poses a high global public health burden, with 249 million malaria cases in 2022 globally [1]. It is a potentially life-threatening disease caused by *Plasmodium* parasites, primarily transmitted to humans through the bites of infected female *Anopheles* mosquitoes.

Healthcare-associated infections (HAIs), known as nosocomial infections, refer to infections acquired by patients during their stay in a hospital or another healthcare setting. These infections are not present or incubating at the time of admission but are acquired within the healthcare environment. Bacteria and viruses are the most common HAI agents, but parasitic protozoa can also cause HAIs.

While there have been sporadic reports of healthcare-associated malaria (HAM) cases and outbreaks in the healthcare setting [2], there is no comprehensive overview of such infections, limiting our knowledge on the frequency and cause of these events. This systematic review aims to explore the epidemiological, clinical and biological determinants of HAM cases. By reviewing the available evidence, this study also aims to provide valuable insights for the development of guidance and protocols to prevent HAM.

## Methods

This systematic review follows the Preferred Reporting Items for Systematic Reviews and Meta-Analyses (PRISMA) guidelines [3] and was registered in PROSPERO (CRD42022309701). Risks of bias were assessed using the Risk of Bias in Systematic Reviews tool [4].

### Definitions

The following definitions are used in this study. A primary case is a malaria case that introduces the *Plasmodium* protozoa into the healthcare facility. A secondary case is a HAM case with an epidemiological link and/or microbiological link with the primary case. A tertiary case is a HAM case with an epidemiological and/or microbiological link with a secondary case. A cluster is a group of cases that are epidemiologically and/or microbiologically linked, composed of the primary case and one or more HAM cases. Iatrogenic transmission is the transmission of a pathogen caused or facilitated by medical procedures, interventions or treatments. We excluded from this definition the donor-derived infection (e.g. through blood transfusion). An epidemiological link is established when there is a coincidence in time, location, contact with the same healthcare personnel and/or use of the same medical equipment or materials. A microbiological link is established when *Plasmodium* strains show similar genotyping and/or sequencing results.

### Searches

On 22 April 2022, we searched PubMed, EMBASE.com, Scopus, OpenGrey and ProQuest for studies on protozoan HAI published since 1 January 2000 (Box). The keywords (MeSH terms) are presented in Box. Further details of the searches are available in Supplementary Table S1. No language or document type restriction was applied. On 7 December 2023, we ran a complementary search to identify any recent publication on HAM exclusively; the complementary search is presented in Supplementary Table S2.

Box **Keywords (MeSH terms) used to search PubMed, EMBASE.com, Scopus, OpenGrey and ProQuest for studies on protozoan healthcare-associated infections published since 1 January 2000**

‘Cross Infection’, ‘Leishmaniasis’, ‘Chagas Disease’, ‘Trypanosomiasis, African’, ‘Giardiasis’, ‘Coccidiosis’, ‘Sarcocystosis’, ‘Isosporiasis’, ‘Toxoplasmosis’, ‘Cryptosporidiosis’, ‘Cyclosporiasis’, ‘Isosporiasis’, ‘Babesiosis’, ‘Amebiasis’, ‘Malaria’, ‘Plasmodium’, and ‘Protozoan Infections’.

The full search strategy is available in the Supplementary Tables S1 and S2.

We performed a backward and forward search on five articles identified through the database search. These articles were selected based on their representativeness, as determined by the authors' judgment. Additionally, we searched over 70 websites of regional and national public health authorities in 45 countries on five continents (Africa: two; America: four; Asia: five; Europe: 32; Oceania: two) and of international health organisations for published case reports. This website list is presented in Supplementary Material S1. We also performed a Google search with keywords (based on MeSH terms) and searched the abstracts of the European Scientific Conference on Applied Infectious Disease Epidemiology (ESCAIDE) conference for years 2009 (earliest date available) to 2023 [5]. These searches were designed to capture data on protozoan HAI, encompassing a broader scope than HAM. Initially, the systematic review aimed to cover protozoan HAI comprehensively. However, due to the limited data on protozoan infections beyond malaria in healthcare settings, this article focuses solely on HAM.

Furthermore, in July 2023, a data call for HAM cases (non-donor derived) detected since 2000 was sent to national public health institutes in 37 European countries (country list provided in the Supplementary Material S2).

### Screening of the literature

Citations identified through the database searches were imported into EndNote for de-duplication and transferred into Rayyan for screening [6]. Two reviewers (CMG and LKHW) independently conducted a first screen by title and abstract. Articles were included when the cases were classified as HAI by the investigators and the full text retrieved. Articles were excluded if transmission was donor-derived, transmission was from mother to child during delivery without the intervention of healthcare personnel, transmission occurred prior to hospital admission or no infection of humans occurred (only findings in animals or the environment).

Two reviewers (CMG and LKHW) independently performed a second screen on the full text articles. The articles not presenting original data were excluded but their references were screened to identify relevant citations.

Disagreements during the two screening stages were resolved by consensus together with a third reviewer (HN). A list of the articles excluded due to not meeting the eligibility criteria or being duplicates is available in Supplementary Table S3.

### Quality assessment and data extraction from the literature

The quality assessment criteria were adapted from the National Institutes of Health Quality Assessment Tool for case series studies [7], and included a series of seven questions related to the clarity and precision of the case information and to the evaluation of possible alternative routes of transmission. The quality assessment criteria are described in Supplementary Table S4. For articles presenting multiple cases, we added an eighth question related to the individual assessment of the route of transmission for each case. We attributed one point per criterion met and articles were assessed as follows: 0 to 3 points = poor quality; 4 to 5 points = fair quality; 6 to 8 points = good quality. No articles were excluded due to poor quality.

The first reviewer (CMG) assessed the quality of the included full-text articles and a random 10% of the articles were checked by a second reviewer (LKHW). One reviewer (CMG) extracted the data, which were checked by one of the two other reviewers (LKHW, HN). Disagreement in the extracted data was resolved by consensus with all reviewers.

After data extraction, cases were deduplicated based on the place and date of infection and case characteristics to identify cases described multiple times.

### European malaria surveillance data query

We queried The European Surveillance System (TESSy) of the European Centre for Disease Prevention and Control for malaria surveillance data to capture complementary information on the HAM cases already identified through the searches. In TESSy, data were available from 27 European Union countries, Iceland, Liechtenstein, Norway and the United Kingdom (UK) (until 2019) for the period 2008–2022.

### Data cleaning and analysis

We used Microsoft Excel to perform a descriptive analysis of the HAM cases data. The main outcomes assessed were the occurrence of (multiple) secondary cases, the occurrence of tertiary cases and death in secondary/tertiary cases.

We estimated the incubation period using the number of days between the (suspected) dates of infection and symptom onset. For cases with multiple potential dates of infection, we used the median date.

For HAM, when percentage of parasitaemia was not provided but the number of trophozoites of *Plasmodium* per μl was available, we estimated the percentage using the following formula, with 5 million cells per μl as the standard value for the total red blood cell count:


$$Percentage\;Parasitaemia=\frac{Number\;of\;trophozoites}{Total\;red\;blood\;cell\;count\;}\;x\;100$$

Hyperparasitaemia was defined as a parasitaemia ≥ 5%.

For ordinal values, we reported medians and interquartile ranges (IQR) (25^th^ and 75^th^ percentiles). A Mann–Whitney U-tests test was used to compare continuous variables with statistical significance assessed at the 0.05 level.

## Results

**Figure 1:**
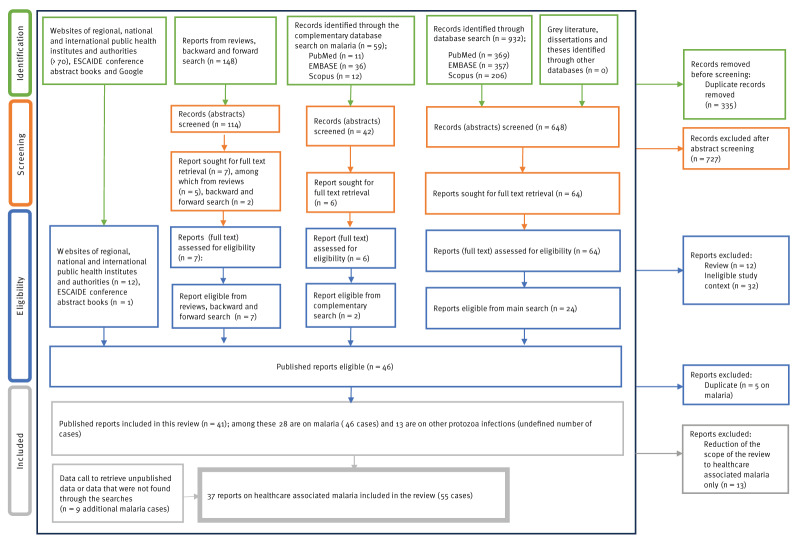
Flowchart describing the process of the literature search

Twenty-four eligible articles were identified through database searches, with an additional two studies found through the complementary malaria search (Figure 1).

ESCAIDE: European Scientific Conference on Applied Infectious Disease Epidemiology.

Furthermore, 20 eligible studies were identified through reviews, backward and forward searches, websites of regional, national and international public health institutes and authorities and the ESCAIDE abstract books. The data call on malaria yielded nine additional malaria cases, including two previously unpublished. After removing duplicates and studies that were on protozoan HAI other than malaria, 37 studies on malaria remained (55 cases) [2,8-37] (personal communication, Milda Žygutienė, 25 August 2023). The list of publications included, and the data extracted are available in Supplementary Table S5.

The majority of the HAM reports were in English (n = 23), followed by Spanish (n = 6), French (n = 1) and German (n = 1). Six reports were not published. Among the published reports, 17 were rated as good [2,9,10,12,16,17,19-24,27,30-33], five as fair [8,28,29,36,37] and nine poor [11,13-15,25,26,34,35,38]. Detailed results on the quality assessment are presented in Supplementary Table S6.

**Figure 2:**
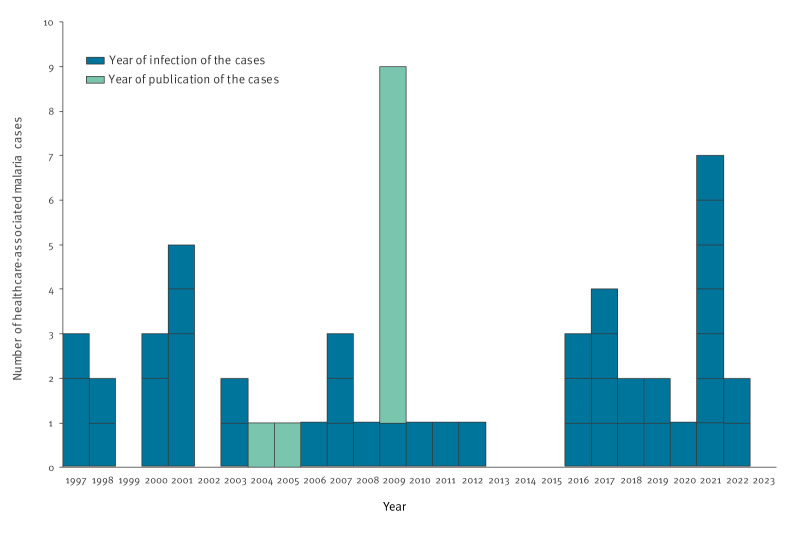
Number of healthcare-associated malaria cases per year of infection (n = 45), year of publication (n = 10) and per cluster (n = 42), 1997–2023

Published case information came from 19 peer-reviewed articles (35 cases), 11 epidemiological bulletins (13 cases), 1 conference presentation (1 case), all of which were case reports (Table 1). Cases were infected between 1997 and 2022. On average, there were 1.7 cases reported each year, with peaks in 2001 (five cases) and 2021 (seven cases) (Table 1; Figure 2). For 10 cases, only the year of publication was available (2004, 2005 and 2009).

**Table 1:** Characteristics of healthcare associated malaria cases and mode of transmission, per cluster, 1997–2022 (n = 55)

Country	Year of infection	Number of cases	Plasmodium species	Sex and age group	Ward of infection	Mode of transmission	Suspected or confirmed procedure leading to infection	Suspected or confirmed vehicle of transmission	Comments	References or sources
Brazil	1998	1	*Plasmodium falciparum*	0–10 years M	Not specified	Iatrogenic	Not specified	Not specified	Patient infected from an imported case	[21]
Colombia	2001	3	*P. falciparum*	11–20 years M; 41–50 years M; 41–50 years M	Emergency ward for the first case; subsequent transmissions were within the internal medicine ward	Iatrogenic	Flushing of vascular catheter	Heparin/saline solution	One secondary case infected from an imported case; two tertiary cases infected from the secondary case. All were hospital patients	[16]
France	2001	1	*P. falciparum*	21–30 years F	Not specified	Iatrogenic	Manipulation of intravenous catheter other than flushing	Needle	HCW infected from an imported case	[29]
France	2021	1	*P. falciparum*	0–10 years F	Emergency ward	Iatrogenic	Manipulation of intravenous catheter other than flushing	Unknown	Patient infected from an imported case	[37]
France	2007	1	*P. falciparum*	0–10 years F	Emergency ward	Iatrogenic	Unknown	Unknown	Patient infected from an imported case	[32]
France	2019	1	*P. falciparum*	71–80 years F	Emergency ward	Iatrogenic	Capillary blood glucose measurement	Unknown	Patient infected from an imported case	[32]
France	2021	1	*P. falciparum*	71–80 years M	Emergency ward	Iatrogenic	Unknown	Unknown	Patient infected from an imported case	[32]
France	2021	1	*P. falciparum*	71–80 years M	Emergency ward	Iatrogenic	Unknown	Unknown	Patient infected from an imported case	[32]
France	2021	1	*P. falciparum*	41–50 years M	Intensive care unit	Iatrogenic	Manipulation of intravenous catheter other than flushing	Unknown	Patient infected from an imported case	[32]
Germany	2007	1	*P. falciparum*	F	Obstetrics ward	Iatrogenic	Unknown	Unknown	HCW infected from an imported case	[31]
Germany	2016	1	*P. falciparum*	31–40 years F	Not specified	Iatrogenic	Unknown	Unknown	Patient infected from an imported case	[17]
Germany	2017	1	*P. falciparum*	21–30 years F	Not specified	Iatrogenic	Unknown	Needle	HCW infected from imported case	[34]
Greece	2021	3	*P. falciparum*	41–50 years F; 61–70 years M; 51–60 years F	COVID-19 unit	Iatrogenic	Unknown	Unknown	Patients infected from an imported case	[26]
Greece	2017	1	*P. falciparum*	31–40 years M	Internal medicine ward	Iatrogenic or vector-borne	Unknown, most likely capillary blood glucose measurement or flushing of vascular catheter or other manipulation of intravenous catheter (if iatrogenic)	Unknown	Patient infected from an imported case	[2,18]
Greece	2020	1	*P. falciparum*	41–50 years F	Internal medicine ward	Iatrogenic or vector-borne	Unknown, most likely flushing of vascular catheter or other manipulation of intravenous catheter (if iatrogenic)	Unknown	Patient infected from an imported case	[25]
Italy	2000	1	*P. falciparum*	51–60 years M	Intensive care unit	Iatrogenic	Glycaemia measurement	Glucometer	Patient infected from an imported case	[24]
Italy	2003	1	*P. falciparum*	Not specified	Not specified	Iatrogenic	Unknown	Unknown	Patient infected from an imported case	Supplementary Table S5
Italy	2007	1	*P. falciparum*	Not specified	Not specified	Iatrogenic	Unknown	Unknown	Patient infected from an imported case	Supplementary Table S5
Italy	2008	1	*P. falciparum*	Not specified	Not specified	Iatrogenic	Unknown	Unknown	Patient infected from an imported case	Supplementary Table S5
Italy	2009	1	*P. falciparum*	Not specified	Not specified	Iatrogenic	Unknown	Unknown	Patient infected from an imported case	Supplementary Table S5
Italy	2017	1	*P. falciparum*	11–20 years M	Emergency ward	Iatrogenic	Not specified	Not specified	Patient infected from an imported case	[2,12]
Italy	2017	1	*P. falciparum*	0–10 years F	Paediatric ward	Iatrogenic	Not specified	Not specified	Patient infected from an imported case	[2,12]
Libya	1997	2	*P. falciparum*	41–50 years F; 60 years F	Infectious Disease unit	Iatrogenic	Manipulation of intravenous catheter other than flushing	Gloves	Patient infected from an imported case	[27]
Lithuania	2016	1	*P. falciparum*	21–30 years F	Emergency ward	Iatrogenic	Not specified	Needle	HCW infected from an imported case	Personal communication, Milda Žygutienė, 25 August 2023
Saudi Arabia	1997	1	*P. falciparum*	0–10 years F	Paediatric ward	Iatrogenic	Flushing of cannula	Heparin/saline solution	Patient infected from an imported case	[9]
Saudi Arabia	Prior to 2010	8	*P. falciparum*	7 cases between 0 and 10 years; 1 case with unknown age	Paediatric ward	Iatrogenic	Flushing of cannula	Heparin/saline solution	Patient infected from an imported case	[8]
South Korea	2006	1	*P. falciparum*	51–60 years M	Emergency ward	Iatrogenic	Unknown	Unknown	Patient infected from an imported case	[20]
Spain	2010	1	*P. falciparum*	61–70 years M	Not specified	Not specified	Not specified	Not specified	Patient infected from an imported case	[13,15]
Spain	2016	1	*P. falciparum*	61–70 years M	Emergency ward	Iatrogenic	Not specified	Not specified	Patient infected from an imported case	[2,15]
Spain	1998	1	*P. falciparum*	61–70 years M	Reanimation ward	Not specified	Not specified	Not specified	Patient infected from an imported case	[28]
Spain	2018	1	*P. malariae and P. ovale*	0–10 years F	Not specified	Iatrogenic	Unknown	Unknown	Patient infected from an imported case	[2]
Spain	2011	1	*P. falciparum*	0–10 years M	Not specified	Not specified	Not specified	Not specified	Patient infected from an imported case	[14,15]
Spain	2022	1	*P. falciparum*	41–50 years M	Not specified	Iatrogenic	Unknown	Unknown	Patient infected from an imported case	[33]
Spain	2018	1	*P. falciparum*	71–80 years M	Not specified	Iatrogenic	Unknown	Unknown	Patient infected from an imported case	[35]
Spain	2019	1	*P. falciparum*	71–80 years M	Internal medicine ward	Iatrogenic	Unknown	Unknown	Patient infected from an imported case	[36]
Spain	2022	1	*P. falciparum*	71–80 years M	Not specified	Iatrogenic	Unknown	Unknown	Patient infected from an imported case	Supplementary Table S5
Netherlands	Unknown	1	*P. falciparum*	11–20 years M	Paediatric ward	Iatrogenic	Unknown	Unknown	Patient infected from an imported case	[30]
UK	2001	1	*P. falciparum*	‘Elderly’ M	Surgical ward	Iatrogenic	Unknown	Unknown	Patient infected from a HCW	[11,50]
UK	Unknown	1	*P. falciparum*	41–50 years F	Infectious disease unit	Iatrogenic	Manipulation of intravenous catheter other than flushing	Unknown	Patient infected from an imported case	[23]
US	2003	1	*P. falciparum*	11–20 years F	Paediatric ward	Iatrogenic	Flushing of cannula	Heparin/saline solution	Patient infected from an imported case	[19]
US	2000	2	*P. falciparum*	51–60 years F; 71–80 years M	Not specified for the HCW and emergency war for the patient	Iatrogenic	Manipulation of intravenous catheter other than flushing	Needle stick injury for HWC and hands with micro-bleeding for the patient	HCW infected from an imported case and Patient infected from a HCW	[10]
US	2012	1	*P. falciparum*	41–50 years F	Surgical ward	Iatrogenic	Glycaemia measurement	Glucometer	Patient infected from an imported case	[22,51]

F: Female; HCW: healthcare-worker; M: Male; UK: United Kingdom; US: United States.

Unspecified: the authors did not mention it; Unknown: the authors specified it remained unknown, despite investigations.

The 55 cases belonged to 42 clusters, with an average cluster size of 1.3 cases. The largest cluster included eight children infected in a paediatric ward in Saudi Arabia [8].

Most reports were on cases (n = 35; 64%) infected in Europe: Spain (n = 9; nine clusters) [2,13-15,28,33,35,36], France (n = 7; seven clusters) [29,32,37], Italy (n = 7; seven clusters) [12,24], Greece (n = 5; three clusters) [18,25,26], Germany (n = 3; three clusters) [17,31,34], the UK (n = 2; two clusters) [11,23], Lithuania (n = 1) (personal communication, Milda Žygutienė, 25 August 2023) and the Netherlands (n = 1) [30]. The remaining cases were reported from infections in Saudi Arabia (n = 9; two clusters) [8,9], the United States (n = 4; three clusters) [10,19,22], Colombia (n = 3; one cluster) [16], Libya (n = 2; one cluster) [27], Brazil (n = 1) [21] and in South Korea (n = 1) [20]. Through the data call, 23 European countries reported no detection of HAM since 2000: Albania, Austria, Belgium, Bulgaria, Cyprus, Czechia, Estonia, Finland, Hungary, Ireland, Kosovo**^‡^**, Latvia, Liechtenstein, Luxembourg, Malta, Montenegro, North Macedonia, Norway, Portugal, Romania, Slovakia, Slovenia and Sweden.

Fifty HAM cases (91%) reported no travel to a malaria endemic country prior infection. No information was available for the remaining cases.

**Figure 3:**
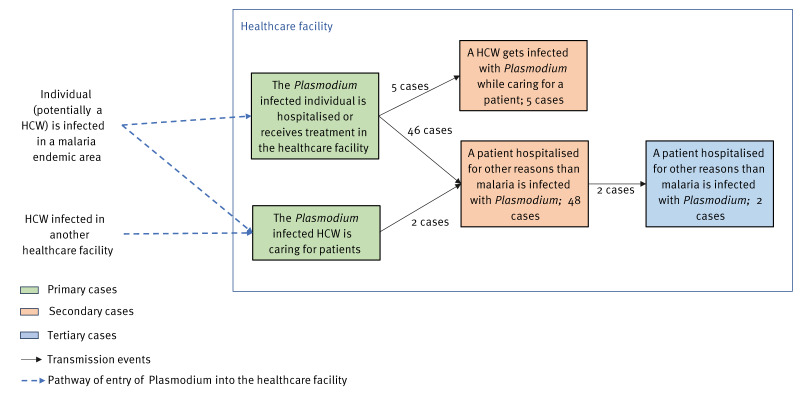
Transmission pathways of *Plasmodium* in the healthcare setting

Of the 55 HAM cases, 54 were infected with *Plasmodium falciparum* and one case was co-infected with *Plasmodium malariae* and *Plasmodium ovale*. Overall, 50 HAM cases were hospital inpatients and five were healthcare workers (HCWs) [10,29,31,34] (personal communication, Milda Žygutienė, 25 August 2023). For two of the patients, the primary case was a HCW [10,11] (Figure 3). Fifty-three HAM cases were secondary cases (patient or HCW), and two inpatients were tertiary cases.

HCW: healthcare worker.

Information on age and sex was available for 44 and 38 patients, respectively. The median age of patients was 41 years (IQR: 5–61); 23 of the patients were men. The five HCWs were female. Age was available for four HCWs, with median age 27 years (IQR: 25–35). Among the 34 cases with known outcome, five died (case fatality rate: 15%); all were patients.

Patients were infected in paediatric wards (n = 12 cases; five clusters), emergency departments (n = 10; 10 clusters), internal medicine wards (n = 5; four clusters), infectious diseases units (n = 3; two clusters), COVID-19 unit (n = 3; one cluster), surgical wards (n = 2; two clusters), intensive care units (n = 2; two clusters), or resuscitation area (n = 1). Healthcare workers were infected in an obstetric ward (n = 1) and an emergency department (n = 1). For twelve patients (12 clusters) and three HCWs (three clusters), the ward of infection was not provided.

An epidemiological link to a primary case could be identified or suspected for 52 HAM cases (39 clusters). No information was provided for the remaining three cases and clusters [12]. For 25 HAM cases (23 clusters), the epidemiological link was supported by a microbiological link, using genotyping and/or sequencing methods. For three cases in one cluster, the molecular typing remained inconclusive [15] and for the rest, either no information was provided, or no samples were retrieved.

Thirty of the primary cases were infected during travel in a malaria-endemic country or region: Cameroon (four primary cases), Nigeria (n = 4), Côte d’Ivoire (n = 3), Equatorial Guinea (n = 3), Burkina Faso (n = 2), The Gambia (n = 2), Africa (unspecified country, n = 1), Brazil (n = 1), Colombia (n = 1), Democratic Republic of the Congo (n = 1), Kenya (n = 1), Mali (n = 1), Mauritania (n = 1), Niger (n = 1), Nigeria or Congo (n = 1), Philippines (n = 1), Senegal (n = 1) and Sudan (n = 1). The primary case co-infected with *P. malariae and P. ovale* was returning from Equatorial Guinea.

For 53 HAM cases, including the five HCWs, the infection was classified as iatrogenic. For two cases in Greece, infection was most likely iatrogenic but vector-borne transmission could not be excluded (despite the fact that for one case the infection - if vector-borne - would have occurred during late November to early December) [18,25].

The procedure leading to infection was described for 24 HAM cases, which were part of 13 clusters. Flushing of a vascular catheter with contaminated heparin/saline solution was the most frequent procedure leading to infection, with 13 cases grouped in four clusters, followed by other manipulation of intravenous catheter (eight cases including two HCWs; six clusters) and capillary blood glucose measurement (three cases; three clusters) (Table 2). When specified, infections during other manipulation of intravenous catheter occurred via contaminated gloves, needles or hands with micro-bleeding. In two instances, HCWs were infected via needlestick injury but the procedures leading to these injuries were not described [34] (personal communication, Milda Žygutienė, 25 August 2023).

**Table 2:** Number of cases and clusters of healthcare-associated malaria among patients and healthcare workers by mode of transmission, 1997–2023 (n = 55)

Mode of infection	Patient	HCW	Total cases	Total clusters	References
Flushing of vascular catheter with contaminated heparin or saline solution	13	0	13	4	[8,9,16,19]
Other manipulation of intravenous catheter	6	2	8	6	NA
Infection via contaminated gloves	2	0	2	1	[27]
Infection via HCW’s hands with micro-bleeding	1^a^	0	1	1^b^	[10]
Needlestick injury during manipulation of intravenous catheter	0	2	2	2^b^	[10,29]
Unknown or not specified	3	0	3	3	[23,32,37]
Capillary blood glucose measurement	3	0	3	3	NA
Glucometer	2	0	2	2	[22,24]
Unknown or not specified	1	0	1	1	[32]
Procedure not specified	7	2	9	9	NA
Needlestick injury during an unspecified procedure	0	2	2	2	[34] (personal communication, Milda Žygutienė, 25 August 2023)
Not specified	7	0	7	7	[12-15,21,28]
Procedure unknown	21	1	22	20	[2,11,17,18,20,25,26,30-33,35,36]

HCW: healthcare worker; NA: not applicable.

^a^ Patient infected from a HCW.

^b ^One cluster included both a transmission via needlestick injury and a transmission via hands with micro-bleeding.

Unknown: investigations were inconclusive; not specified: the study/record did not provide the information.

Among the 47 HAM hospitalised patients for which a primary case was identified or suspected, eight shared the same room as the primary case, nine were in nearby rooms on the same ward, 10 were on the same ward but in different rooms (with no information on the proximity of the rooms), 16 were on the same ward, but specific room information was not available, one was on different wards or floors and no information was available for the remaining case. Additionally, two patients were infected by an HCW.

Among the 25 patients with information about length of co-hospitalisation with the primary or secondary case when applicable, 11 cases were co-hospitalised for 1 day or less (44%), 13 were co-hospitalised for 2 to 7 days (52%), and one was co-hospitalised for more than 7 days (4%). The median time lag between admission to the hospital of the primary case and infection of the secondary case could be calculated for 13 HAM cases and was estimated to be 2 days (IQR: 0.5–2).

Based on 24 HAM cases with information, the median incubation period (IP) was estimated to be 15 days (IQR: 10–20). More specifically, the median IP was 16 days (IQR: 11–20) among patients with *P. falciparum* infection (20 cases with information) and the IP was 21 days for the patient with *P. malariae* and *P. ovale* co-infection. The median IP among the HCWs was 10 days (three cases with information).

The median diagnostic delay (time between symptom onset and diagnosis), estimated based on 33 HAM cases, was 6 days (IQR: 3–10). This delay was 7 days (IQR: 4–10) among patients infected in Europe (estimated for 20 patients) and 10 days among HCW (estimated for three HCW). In comparison, among the 42,292 travel-related malaria cases reported to TESSy, the median diagnostic delay was 4 days (IQR: 2–7). The difference in diagnostic delay between travel-related cases and either all HAM cases or HAM cases infected in Europe was statistically significant (p < 0.05).

Among the 22 HAM cases for which parasitaemia at diagnosis was provided or calculated, 11 had hyperparasitaemia. Six of the hyperparasitaemic HAM cases recovered, one died, and no outcome information was available for the other cases. The median diagnostic delay was 10 days (IQR: 4–11) and 8 days (IQR: 2–14) for HAM cases with hyperparasitaemia and without hyperparasitaemia, respectively. Parasitaemia of the primary cases was provided or calculated for 16 cases, among which eight had hyperparasitaemia. We could not identify any specific patterns between hyperparasitaemia of the primary case with specifics of the HAM cases.

Entomological investigations were carried out for 13 clusters. In one instance, the entomological investigation found larvae of *Anopheles maculipennis s.l.* but no adult mosquitoes were found [31]. No competent vectors were identified during the course of the other entomological investigations. For five clusters there were no entomological investigations - four of them occurred between December and February in Europe and no information about the month of occurrence was provided for the fifth cluster. No information about potential entomological investigations carried out was provided for the remaining 24 clusters.

## Interpretation

Through this systematic review we identified 55 HAM cases with symptom onset between 1997 and 2022. We believe most of the HAM cases have been captured through this systematic review, at least for Europe, where malaria is not endemic but notifiable. The data call allowed us to complement the searches, and importantly, to confirm the absence of identification of HAM cases in most European countries.

The median IP for HAM cases was 15 days, which is on the upper side of the commonly known range of 7 to 15 days and longer than what was previously described in a similar review (12 days) [29]. The IP should be considered with caution, as the date of infection may not have been recorded or identified accurately and the date of symptom onset may be difficult to define in patients who already have other symptoms. The diagnostic delay among HAM cases in Europe was more than twice that observed among European travellers (7 days vs 3 days). A longer diagnostic delay among HAM cases was expected as malaria would not be immediately suspected among people without relevant travel history. Delayed diagnosis increases the likelihood of severe disease and adverse outcomes. Healthcare providers should consider HAM in patients with unexplained fever or malaria-like symptoms, especially if their admission coincided with another patient diagnosed with malaria. Studies have shown that the prevalence of thrombocytopenia is significantly higher among malaria patients compared with those with other acute febrile illnesses [38]. As such, thrombocytopenia could be an additional trigger to early diagnosis of HAM patients.

The majority of HAM were due to *P. falciparum*. First, it is the most common *Plasmodium* species among travel-related cases in Europe [39,40], second there is a higher likelihood for *P. falciparum* cases to be hospitalised [41,42] and third, *P. falciparum* has a high reproductive rate within red blood cells, leading to a higher parasitaemia compared with other *Plasmodium* species [41,43,44]. This makes *P. falciparum*-containing blood particularly infectious, facilitating secondary infections when standard precautions are not strictly applied, even when potentially microscopic traces of blood contaminate medical devices and other objects that are subsequently used in other patients. The inoculation of even a single infected erythrocyte is theoretically considered sufficient for transmission [45,46].

The paediatric ward is where most individuals were infected. This result is driven by one outbreak that occurred in Saudi Arabia with eight children infected due to a contaminated heparin solution [8]. The highest number of clusters occurred in the emergency department. This is compatible with the fact that imported cases with severe malaria are likely treated in this ward and invasive procedures performed even before the diagnosis is considered or confirmed. In addition, we could hypothesise that the medical staff in emergency departments work under high pressure and stress, which could, in rare instances, lead to breaches in infection prevention and control procedures. The increase in number of cases in 2021, a year marked by the COVID-19 pandemic, supports this hypothesis, especially considering that the number of travel-related cases of malaria dropped globally due to the travel restrictions.

The procedure leading to infection could be identified in 13 of 42 clusters and 24 of 55 individuals. Flushing of vascular catheter with contaminated heparin/saline solutions is the procedure that led to the largest cluster size. Other manipulation of intravenous catheters led to the highest number of clusters when infections of HCWs were included.

For most clusters, investigations remained inconclusive with regard to the procedure leading to infection despite extensive investigation, highlighting the complexity of such investigations. Investigations often rely on interviews which have limitations due to recall bias and accountability concerns. Contamination of products, devices and surfaces with microscopic traces of blood that have been implicated in HAM is, in most cases, difficult to confirm due to the unavailability of the contaminated material at the time of diagnosis. In such cases, the exclusion of other modes of transmission makes an unidentified breach in standard precautions the most likely explanation.

In this systematic review, we used a broad definition of HAM, including non-donor derived iatrogenic and mosquito-borne transmission. For two isolated clusters in Greece, investigations concluded that the mode of transmission was either iatrogenic (non-donor derived) or by vectors, although the iatrogenic route was considered the more plausible. Although no *Anopheles* mosquitoes were caught in and around the hospital premises, the presence of such mosquitoes could not be formally excluded. In these instances, the two healthcare facilities were located in peri-urban areas where Anophelinae presence could be expected.

In the other cases, the most common reasons for excluding the hypothesis of mosquito transmission were the absence of competent vectors, during on-site entomological investigations and/or because of unsuitable climate conditions (e.g. winter) at the time of occurrence. Another reason was too short of a time interval between hospitalisation of the primary case and infection of the secondary case. In fact, after an anopheline female takes a blood meal from an infected individual, the parasite goes through a maturation cycle in the mosquito (i.e. extrinsic incubation period), lasting 8 to 35 days for *P. falciparum* [47], which is required to further transmit the parasite. Mosquito transmission of *P. falciparum* could therefore be considered only in cases where there is a delay of at least 15 days (extrinsic incubation period of 8 days [47], plus the shortest intrinsic incubation period in humans for parasite development of 7 days [48]) between hospitalisation of the primary case and onset of symptoms of the secondary case, knowing that the infecting bite of the mosquito might not have taken place on the first day of hospitalisation.

Mechanical transmission by arthropod vectors has not been demonstrated, hence not considered as a transmission pathway.

Occurrence of airport or luggage malaria, importation of infected mosquito from malaria endemic countries, in the healthcare setting cannot be excluded but seems very unlikely. However, such an event was described in a hospital located at proximity to an airport in France in February 1995 [49].

Healthcare workers drawing blood and/or inserting or manipulating vascular access catheters on imported malaria cases are at risk of infection as this procedure could lead to infection through needlestick injury or other contact with contaminated blood. In the event of a needlestick injury from a malaria patient, the affected individual should be advised about the possibility of malaria transmission to facilitate early diagnosis and treatment to prevent adverse outcomes (and onward transmission). Healthcare workers were involved in six clusters: in five clusters the HCW was infected from a patient and in two clusters the HCWs infected the patient. In one event, one HCW was both infected by a patient and transmitted the infection to another patient [10].

This systematic review on healthcare-associated malaria faces several limitations. Single cases with inconclusive findings are often not published due to their lack of novelty, leading to potential underreporting of such instances. Additionally, hospitals have little incentive to publish information on gaps in infection control precautions as it may reflect negatively on their reputation. This further skews the available data, making it challenging to obtain a comprehensive understanding of the issue. To counter these limitations, we conducted a data call to European countries and examined grey literature. Malaria is a mandatory notifiable disease in most European countries, and we expect that most cases have been captured, providing a more accurate representation of the situation.

The geographical distribution of HAM cases is inherently biased by the endemicity level of each country. Healthcare-associated malaria cases are more likely to be identified in areas where malaria is not endemic. Of note, HAM cases from Brazil and Colombia, countries with some high endemicity regions, occurred in areas considered malaria free.

Overall, the quality of the evidence gathered is considered satisfactory (good or fair), with the majority of studies providing detailed information about the investigations. Most studies that scored poorly were annual epidemiological reports, which aimed to provide a general overview of malaria surveillance rather than detailed information about specific investigations.

## Conclusions

While rare, HAM transmission can be fatal. Healthcare-associated malaria is preventable through a strict adherence to infection prevention and control procedures. To minimise any residual risk, specific additional measures can be considered. For instance, procedures involving routine management of intravascular access or capillary blood glucose measurement in patients with malaria should be performed at the end of a ward round and dedicated equipment should be used when possible. In addition, for the prevention of mosquito-borne transmission, mosquito repellents and bed nets should be used in infectious malaria patients in areas with Anophelinae presence. For the rapid diagnosis of HAM cases, HCW should keep in mind the possibility of HAM and rapidly perform a diagnostic test upon any suspicion of HAM. Investigations of HAM transmission events often remain inconclusive, despite extensive investigations. The development of guidance and protocols for conducting such investigations could help improve the success rate of such inquiries. By establishing standardised protocols, healthcare facilities may be better equipped to identify gaps in infection control, implement targeted interventions and ultimately reduce the risk of *Plasmodium* transmission within their settings.

## ^‡^Note

This designation is without prejudice to positions on status and is in line with United Nations Security Council Resolution 1244/99 and the International Court of Justice Opinion on the Kosovo Declaration of Independence.

## Supplementary Data

311000 Supplementary Material

## Supplementary Data

771000 Supplementary Table S5
