# Supplementary material for: Healthcare-associated malaria: a systematic review, 1997 to 2023
Source: Euro Surveill. 2025 Mar 20;30(11):2400393. doi: 10.2807/1560-7917.ES.2025.30.11.2400393 (PMC11927069; doi:10.2807/1560-7917.ES.2025.30.11.2400393)
Supplement: Supplementary Material [file 24-00393_GOSSNER_Supplementary_materials.pdf]

Supplementary material

This supplementary material is hosted by *Eurosurveillance* as supporting information alongside the article *Healthcare-associated malaria: a systematic review, 1997 to 2023*, on behalf of the authors, who remain responsible for the accuracy and appropriateness of the content. The same standards for ethics, copyright, attributions and permissions as for the article apply. Supplements are not edited by *Eurosurveillance* and the journal is not responsible for the maintenance of any links or email addresses provided therein.

Eight supplementary materials are provided:

Supplementary Table S1 presents the database search in PubMed, Embase and Scopus, which was conducted on 22 April 2022.

Supplementary Table S2 presents the complementary database search in PubMed, Embase and Scopus, which was conducted on 7 December 2023.

Supplementary Table S3 lists the articles excluded during the assessment of the eligibility criteria, during the data extraction or after the narrowing of the scope of the review.

Supplementary Table S4 describes the quality assessment criteria and verdict.

Supplementary Table S5 provides the list of the publications included and the data extracted for this review.

Supplementary Table S6 presents the results on the quality assessment of the studies included.

Supplementary material S1 provides a list of the websites of regional and national public health authorities and of international health organisations that have been screened.

Supplementary material S2 presents the list of the countries contacted for the healthcare-associated malaria data call.

Supplementary table S1. Search strategy in PubMed, Embase and Scopus.
The search was performed on 22 April 2022

PubMed		
**Search number**	**Query**	**Results**
11	#10 AND 2000:2023[DP]	326
10	#8 AND #9	520
9	"Cross Infection"[Mesh] OR "cross infect*"[TW] OR "healthcare associated infection"[TW] OR "healthcare associated infections"[TW] OR "health care associated infection"[TW] OR "health care associated infections"[TW] OR "healthcare acquired infection"[TW] OR "healthcare acquired infections"[TW] OR "health care acquired infection"[TW] OR "health care acquired infections"[TW] OR "healthcare related infection"[TW] OR "healthcare related infections"[TW] OR "health care related infection"[TW] OR "health care related infections"[TW] OR "hospital related infection"[TW] OR "hospital related infections"[TW] OR "hospital associated infection"[TW] OR "hospital associated infections"[TW] OR "hospital acquired infection"[TW] OR "hospital acquired infections"[TW] OR "hospital infection*"[TW] OR "healthcare infection*"[TW] OR "health care infection"[TW] OR "health care infections"[TW] OR nosocomial*[TW] OR (((healthcare[TI] OR "health care"[TI] OR hospital*[TI]) AND (associat*[TI] OR acqui*[TI] OR relat*[TI])) AND infect*[TI]) OR (((healthcare[OT] OR "health care"[OT] OR hospital*[OT]) AND (associat*[OT] OR acqui*[OT] OR relat*[OT])) AND infect*[OT])	90,580
8	#1 OR #2 OR #3 OR #4 OR #5 OR #6 OR #7	301,575
7	"Leishmaniasis"[Mesh] OR "Chagas Disease"[Mesh] OR "Trypanosomiasis, African"[Mesh] OR leishmania*[TW] OR leishmanios*[TW] OR "chagas disease*"[TW] OR "sleeping sickness"[TW] OR african trypanosomias*[TW] OR chagas*[TW] OR trypanosoma[TW]	79,423
6	"Giardiasis"[Mesh] OR giardia*[TW] OR lamblias*[TW]	11,925
5	"Coccidiosis"[Mesh] OR "Sarcocystosis"[Mesh] OR "Isosporiasis"[Mesh] OR "Toxoplasmosis"[Mesh] OR "Cryptosporidiosis"[Mesh] OR "Cyclosporiasis"[Mesh] OR "Isosporiasis"[Mesh] OR Coccidi*[TW] OR sarcocyst*[TW] OR isosporias*[TW] OR toxoplasm*[TW] OR cryptosporid*[TW] OR cyclospora[TW] OR cyclosporias*[TW] OR cystoisospor*[TW] OR isospor*[TW]	59,991
4	"Babesiosis"[Mesh] OR babesios*[TW] OR babesia*[TW] OR piroplasm*[TW]	8,099
3	"Amebiasis"[Mesh] OR Amebias*[TW] OR amoebias*[TW] OR amebic abscess*[TW] OR ameba[TW] OR amebic[TW] OR amoeba[TW] OR amoebic[TW] OR balamuthia[TW]	22,000
2	"Malaria"[Mesh] OR "Plasmodium"[Mesh] OR malaria*[TW] OR "remittent fever*"[TW] OR "marsh fever*"[TW] OR paludism*[TW] OR (plasmodi*[TW] NOT (plasmodiophor*[TW])) OR (falciparum[TW]) OR (vivax[TW]) OR (knowlesi[TW]) OR (p. w3 ovale[TW])	117,182
1	"Protozoan Infections"[Mesh:NoExp] OR protozo*[TIAB] OR protozo*[OT]	37,852

Embase		
Search number	Query	Results
11	#10 AND [2000-2023]/py	552
10	#8 AND #9	684
9	'hospital infection'/de OR nosocomial*:ab,ti,kw OR ((cross OR hospital OR hospitals OR healthcare OR 'health care') NEXT/3 infect*):ab,ti OR (((healthcare OR 'health care' OR hospital OR hospitals) NEXT/3 (associat* OR acqui* OR relat*) NEAR/5 infect*):ab,ti)	96,507
8	#1 OR #2 OR #3 OR #4 OR #5 OR #6 OR #7	334,501
7	'leishmaniosis'/de OR 'chagas disease'/de OR 'African trypanosomiasis'/de OR leishmania*:ab,ti OR leishmanios*:ab,ti OR 'sleeping sickness*':ab,ti OR ((chagas* NEXT/3 disease*):ab,ti) OR ((african NEXT/3 trypanosomias*):ab,ti) OR trypanosoma:ab,ti	87,355
6	'giardiasis'/de OR giardia*:ab,ti OR lamblias*:ab,ti	14,544
5	'coccidiosis'/de OR 'sarcocystosis'/de OR 'isosporiasis'/de OR 'toxoplasmosis'/de OR 'cryptosporidiosis'/de OR 'cyclosporiasis'/de OR 'isosporiasis'/de OR coccidi*:ab,ti OR cryptosporid*:ab,ti OR cyclospora:ab,ti OR cyclosporias*:ab,ti OR toxoplasm*:ab,ti OR sarcocyst*:ab,ti OR cystoisospor*:ab,ti OR isospor*:ab,ti	68,460
4	'piroplasmosis'/de OR babesios*:ab,ti OR babesia*:ab,ti OR piroplasm*:ab,ti	9,151
3	'amebiasis'/de OR amebias*:ab,ti OR amoebias*:ab,ti OR 'amebic abscess*':ab,ti OR ameba:ab,ti OR amebic:ab,ti OR amoeba:ab,ti OR amoebic:ab,ti or blamuthia:ab,ti	20,198
2	'malaria'/de OR malaria*:ab,ti OR 'remittent fever*':ab,ti OR 'marsh fever*':ab,ti OR paludism*:ab,ti OR (plasmodi*:ab,ti NOT plasmodiophor*:ab,ti) OR falciparum*:ab,ti OR vivax:ab,ti OR knowlesi:ab,ti OR ((plasmodium OR p.) NEAR/3 ovale)	146,253
1	'protozoal infection'/de OR (protozo* NEAR/5 (disease* OR infect*)):ab,ti	15,355

Scopus		
**Search number**	**Query**	**Results**
10	#9 AND AND ((LIMIT-TO (PUBYEAR , 2022) OR LIMIT-TO (PUBYEAR , 2021) OR LIMIT-TO (PUBYEAR , 2020) OR LIMIT-TO (PUBYEAR , 2019) OR LIMIT-TO (PUBYEAR , 2018) OR LIMIT-TO (PUBYEAR , 2017) OR LIMIT-TO (PUBYEAR , 2016) OR LIMIT-TO (PUBYEAR , 2015) OR LIMIT-TO (PUBYEAR , 2014) OR LIMIT-TO (PUBYEAR , 2013) OR LIMIT-TO (PUBYEAR , 2012) OR LIMIT-TO (PUBYEAR , 2011) OR LIMIT-TO (PUBYEAR , 2010) OR LIMIT-TO (PUBYEAR , 2009) OR LIMIT-TO (PUBYEAR , 2008) OR LIMIT-TO (PUBYEAR , 2007) OR LIMIT-TO (PUBYEAR , 2006) OR LIMIT-TO (PUBYEAR , 2005) OR LIMIT-TO (PUBYEAR , 2004) OR LIMIT-TO (PUBYEAR , 2003) OR LIMIT-TO (PUBYEAR , 2002) OR LIMIT-TO (PUBYEAR , 2001) OR LIMIT-TO (PUBYEAR , 2000)))	206
9	#7 AND #8	
8	TITLE-ABS(hospital-acquired OR hospital-associated OR healthcare-associated OR nosocomial* OR iatrogenic*)	
7	#1 OR #2 OR #3 OR #4 OR #5 OR #6	
6	TITLE-ABS(leishman* OR trypanosom* OR chagas OR "sleeping sickness")	
5	TITLE-ABS(giardia* OR lamblia*)	
4	TITLE-ABS(coccidi* OR cyclospora OR cyclosporias* OR cryptosporid* OR isospor* OR cystoisospor* OR sarcocyst* OR toxoplasm*)	
3	TITLE-ABS(babesios* OR babesia* OR piroplasm*)	
2	TITLE-ABS(amebias* OR amoebias* OR 'amebic abscess*' OR ameba OR amebic OR amoeba OR amoebic or blamuthia)	
1	TITLE-ABS(malaria* OR plasmodi* OR falciparum OR (p. W/3 ovale) OR vivax OR knowlesi)	

Supplementary table S2. Complementary search strategy in PubMed, Embase and Scopus.
The complementary search was performed on 07 December 2023

**PubMed**		
**Search number**	**Query**	**Results**
4	#3 AND 2022:2023[DP]	11
3	#1 AND #2	194
2	Cross Infection[Mesh] OR "cross infect*"[TW] OR "healthcare associated infection"[TW] OR "healthcare associated infections"[TW] OR "health care associated infection"[TW] OR "health care associated infections"[TW] OR "healthcare acquired infection"[TW] OR "healthcare acquired infections"[TW] OR "health care acquired infection"[TW] OR "health care acquired infections"[TW] OR "healthcare related infection"[TW] OR "healthcare related infections"[TW] OR "health care related infection"[TW] OR "health care related infections"[TW] OR "hospital related infection"[TW] OR "hospital related infections"[TW] OR "hospital associated infection"[TW] OR "hospital associated infections"[TW] OR "hospital acquired infection"[TW] OR "hospital acquired infections"[TW] OR "hospital infection*"[TW] OR "healthcare infection*"[TW] OR "health care infection"[TW] OR "health care infections"[TW] OR nosocomial*[TW] OR (((healthcare[TI] OR "health care"[TI] OR hospital*[TI]) AND (associat*[TI] OR acqui*[TI] OR relat*[TI])) AND infect*[TI]) OR (((healthcare[OT] OR "health care"[OT] OR hospital*[OT]) AND (associat*[OT] OR acqui*[OT] OR relat*[OT])) AND infect*[OT])	96,654
1	Malaria[Mesh] OR "Plasmodium"[Mesh] OR malaria*[TW] OR "remittent fever*"[TW] OR "marsh fever*"[TW] OR paludism*[TW] OR (plasmodi*[TW] NOT (plasmodiophor*[TW])) OR (falciparum[TW]) OR (vivax[TW]) OR (knowlesi[TW]) OR (p. w3 ovale[TW])	124,644

Embase

Search number	Query	Results
4	#3 AND [2022-2023]/py	36
3	#1 AND #2	343
2	'hospital infection'/de OR nosocomial*:ab,ti,kw OR ((cross OR hospital OR hospitals OR healthcare OR 'health care') NEXT/3 infect*):ab,ti OR (((healthcare OR 'health care' OR hospital OR hospitals) NEXT/3 (associat* OR acqui* OR relat*) NEAR/5 infect*):ab,ti)	104,830
1	'malaria'/de OR malaria*:ab,ti OR 'remittent fever*':ab,ti OR 'marsh fever*':ab,ti OR paludism*:ab,ti OR (plasmodi*:ab,ti NOT plasmodiophor*:ab,ti) OR falciparum*:ab,ti OR vivax:ab,ti OR knowlesi:ab,ti OR ((plasmodium OR p.) NEAR/3 ovale)	156,394

Scopus

Search number	Query	Results
4	#3 AND AND PUBYEAR > 2021 AND PUBYEAR < 2024	12
3	#1 AND #2	158
2	TITLE-ABS(hospital-acquired OR hospital-associated OR healthcare-associated OR nosocomial* OR iatrogenic*)	110,545
1	TITLE-ABS(malaria* OR plasmodi* OR falciparum OR (p. W/3 ovale) OR vivax OR knowlesi)	139,347

Supplementary table S3. Articles excluded during the assessment of the eligibility criteria, during the data extraction or after the narrowing of the scope of the review

First author	Title	Publication year	Journal	Reason exclusion	Timing of exclusion
Lee J.	Intestinal parasite infections at an institution for the handicapped in Korea	2000	The Korean Journal of Parasitology	There is no indication that this centre for handicaped is medicalised and therefore this article does not meet the criteria "healthcare associated"	Excluded during assessment of eligibilty criteria
Squier C.	Waterborne nosocomial infections	2000	Current Infectious Disease Reports	No description of healthcare associated protozoan infection	Excluded during assessment of eligibilty criteria
Bonnet, E.	[Nosocomial diarrhea in adults due to microorganisms other than Clostridium difficile]	2000	Pathologie Biologie	Review; does not present cases of healthcare associated protozoan infection	Excluded during assessment of eligibilty criteria
-	Needle stick injuries: nurses at risk	2000	The Michigan nurse	No description of healthcare associated protozoan infection	Excluded during assessment of eligibilty criteria
Dunn D. L.	Hazardous crossing: immunosuppression and nosocomial infections in solid organ transplant recipients	2001	Surgical Infections	Review; does not present cases of healthcare associated protozoan infection	Excluded during assessment of eligibilty criteria
Weber D.J.	The emerging nosocomial pathogens cryptosporidium, escherichia coli O157:H7, helicobacter pylori, and hepatitis C: Epidemiology, environmental survival, efficacy of disinfection, and control measures	2001	Infection Control and Hospital Epidemiology	Review; does not present cases of healthcare associated protozoan infection	Excluded during assessment of eligibilty criteria
Weber D.J.	Cryptosporidiosis	2002	New England Journal of Medicine	No description of healthcare associated protozoan infection	Excluded during assessment of eligibilty criteria
Saïssy J.M.	L'accès palustre en réanimation	2002	Medecine Therapeutique	Review; does not present cases of healthcare associated protozoan infection	Excluded during assessment of eligibilty criteria
GOV.UK	Cryptic malaria cases in England – 2002	2003	GOV.UK website	Duplicate with Asgari et al.	Excluded during data extraction (duplicates)
Al-Homrany M	Epidemiology of acute renal failure in hospitalized patients: Experience from southern Saudi Arabia	2003	Eastern Mediterranean Health Journal	No data on hospital-acquired infections per pathogen are provided	Excluded during assessment of eligibilty criteria
Tejero R.	[Nosocomial fever of unknown origin in a patient with polytrauma]	2003	Enfermedades Infecciosas y Microbiología Clínica	Donor-derived infection	Excluded during assessment of eligibilty criteria
Virdi V.S.	Neonatal transfusion malaria requiring exchange transfusion	2003	Annals of Tropical Paediatrics	Donor-derived infection	Excluded during assessment of eligibilty criteria
El-Mahallawy H. A.	Epidemiologic Profile of Symptomatic Gastroenteritis in Pediatric Oncology Patients Receiving Chemotherapy	2004	Pediatric Blood and Cancer	No description of healthcare associated protozoan infection	Excluded during assessment of eligibilty criteria

Eliades MJ.	Malaria surveillance--United States, 2003	2005	Morbidity and mortality weekly report	Duplicate with Jain et al.	Excluded during data extraction (duplicates)
Exner M.	Prevention and control of health care-associated waterborne infections in health care facilities	2005	American Journal of Infection Control	No description of healthcare associated protozoan infection	Excluded during assessment of eligibilty criteria
Czartoski T.	Central nervous system infections in transplant recipients	2006	Continuum: Lifelong Learning in Neurology	No description of healthcare associated protozoan infection	Excluded during assessment of eligibilty criteria
Domínguez-Berjón M. F.	[Outbreak of Giardia lamblia in a mother-child institution]	2006	Medicina Clínica	Infection acquired outside the healthcare setting	Excluded during assessment of eligibilty criteria
Sanad M.M.	Cryptosporidiosis among immunocompromised patients in Saudi Arabia	2007	Journal of the Egyptian Society of Parasitology	No clear evidence it is nosocomial infection	Excluded during assessment of eligibilty criteria
Vonberg R.P.	Hospital-acquired infections related to contaminated substances	2007	Journal of Hospital Infection	Review	Excluded during assessment of eligibilty criteria
Boye C. S.	Nosocomial infections: main causative agents	2007	Dakar médical	Review; does not present cases of healthcare associated protozoan infection	Excluded during assessment of eligibilty criteria
Tarantola A.	A textbook case of autochthonous malaria, France, 2007	2009	Escaide Abstract Book	Duplicate with Coppee et al.	Excluded during data extraction (duplicates)
Bobo L	Recognition and prevention of hospital-associated enteric infections in the intensive care unit	2010	Critical Care Medicine	No description of healthcare associated protozoan infection	Excluded during assessment of eligibilty criteria
Sánchez-Lázaro I.J.	Timing, etiology, and location of first infection in first year after heart transplantation	2010	Transplantation Proceedings	No description of healthcare associated protozoan infection	Excluded during assessment of eligibilty criteria
Feasey N.	Keep it clean: Hospital-acquired infections in children	2011	The Lancet	No description of healthcare associated protozoan infection	Excluded during assessment of eligibilty criteria
Ben Abda I	Cryptosporidium infection in patients with major histocompatibility complex class II deficiency syndrome in Tunisia: Description of five cases	2011	Archives de Pediatrie	No description of healthcare associated protozoan infection	Excluded during assessment of eligibilty criteria
Azira N.M.S.	Case series of naturally acquired Plasmodium knowlesi infection in a tertiary teaching hospital	2012	Tropical Biomedicine	Infection acquired outside the healthcare setting	Excluded during assessment of eligibilty criteria
Can ÖS	A Trojan horse in intensive care unit: Toxoplasma gondii	2012	Turkiye Klinikleri Journal of Medical Sciences	Infection acquired outside the healthcare setting	Excluded during assessment of eligibilty criteria
-	IMSACON 2012	2012	Journal International Medical Sciences Academy	No description of healthcare associated protozoan infection	Excluded during assessment of eligibilty criteria
Baijal R	Infections in Cirrhosis of Liver : A multicentre observational study from India	2013	Journal of Gastroenterology and Hepatology	No data on hospital-acquired infections per pathogen are provided	Excluded during assessment of eligibilty criteria
Bousbia S	Serologic Prevalence of Amoeba-Associated Microorganisms in Intensive Care Unit Pneumonia Patients	2013	PLoS ONE	No description of healthcare associated protozoan infection	Excluded during assessment of eligibilty criteria

Cullen K.A.	Malaria surveillance--United States, 2012	2014	Morbidity and mortality weekly report	Duplicate with Lee et al.	Excluded during data extraction (duplicates)
Del Pilar Hernandez M	Infectious complications after liver transplantation	2015	Gastroenterology and Hepatology	Review; does not present cases of healthcare associated protozoan infection	Excluded during assessment of eligibilty criteria
Halder A.	Changing perspectives of infectious causes of maternal mortality	2015	Journal of the Turkish German Gynecology Association	No description of healthcare associated protozoan infection	Excluded during assessment of eligibilty criteria
Ahmad Saleh A. M.	Malaria: A General Minireview with Reference to Egypt	2016	Journal of the Egyptian Society of Parasitology	Review	Excluded during assessment of eligibilty criteria
Ahmad Saleh A. M.	Occupational, Nosocomial or Hospital Acquired Toxoplasmosis	2016	Journal of the Egyptian Society of Parasitology	Review	Excluded during assessment of eligibilty criteria
Brunet J	Ruling out nosocomial transmission of Cryptosporidium in a renal transplantation unit: Case report	2016	BMC Infectious Diseases	Nosocomial transmission excluded	Excluded during assessment of eligibilty criteria
Bammigatti C.	Healthcare associated infections in a resource limited setting	2017	Journal of Clinical and Diagnostic Research	No description of healthcare associated protozoan infection	Excluded during assessment of eligibilty criteria
Cohen B	Transmission of health care-associated infections from roommates and prior room occupants: A systematic review	2017	Clinical Epidemiology	Review	Excluded during assessment of eligibilty criteria
Velasco E.	Non-imported malaria in non-endemic countries: A review of cases in Spain	2017	Malaria Journal	Review	Excluded during assessment of eligibilty criteria
Essid R.	Polymorphism study of Cryptosporidium hominis gp60 subtypes circulating in Tunisia	2017	Microbial Pathogenesis	No clear evidence it is nosocomial infection	Excluded during assessment of eligibilty criteria
Istituto Superiore di Sanità	Malaria - Archivio 2016-2017	2017	NA	Duplicate with Boccolini et al.	Excluded during data extraction (duplicates)
Almuedo-Riera A.	Nosocomial malaria in a splenectomised patient after heart transplantation	2019	Transactions of the Royal Society of Tropical Medicine and Hygiene	Donor-derived infection	Excluded during assessment of eligibilty criteria
Alibakhshi A	The development and evaluation of a multi-epitope antigen as a serodiagnostic marker of Toxoplasma gondii infection	2020	Advances in Clinical and Experimental Medicine	No description of healthcare associated protozoan infection	Excluded during assessment of eligibilty criteria
Fürnkranz U.	Nosocomial infections: Do not forget the parasites!	2021	Pathogens	Review	Excluded during assessment of eligibilty criteria
Mace K.E.	Malaria Surveillance - United States, 2017	2021	Morbidity and mortality weekly report	No description of healthcare associated protozoan infection	Excluded during assessment of eligibilty criteria
Bennett WN	Infectious Disease Teleconsultation to the Deployed U.S. Military From 2017-2022	2022	Mil Med	No description of healthcare associated protozoan infection	Excluded during assessment of eligibilty criteria
Mace KE	Malaria Surveillance - United States, 2018	2022	MMWR Surveill Summ	No description of healthcare associated protozoan infection	Excluded during assessment of eligibilty criteria

de Roquetaillade C	Cytokine profiles in adults with imported malaria	2023	Sci Rep	No description of healthcare associated protozoan infection	Excluded during assessment of eligibilty criteria
Bassat Q	Causes of Death Among Infants and Children in the Child Health and Mortality Prevention Surveillance (CHAMPS) Network	2023	JAMA Netw Open	No description of healthcare associated protozoan infection	Excluded during assessment of eligibilty criteria
Hamad A	Assessment of the incidence and etiology of nosocomial diarrhea in a medical ward in Iraq	2022	Journal of medicine and life	Out of the final scope of the review (i.e. on healthcare associated protozoa infection other than malaria)	Excluded after narrowing the scope of the review (focus on healthcare-associated malaria)
Aguiar A.	Eosinofilia em recém-nascido: Um caso de giardíase e alergia à proteína do leite de vaca [Eosinophilia in a newborn: A case of giardiasis and milk allergy].	2011	Acta Med Port.	Out of the final scope of the review (i.e. on healthcare associated protozoa infection other than malaria)	Excluded after narrowing the scope of the review (focus on healthcare-associated malaria)
Östan İ.	Manisa yöresinde nozokomi yal bağırsak parazitozlarının araştırılması.	2004	Turkiye Parazitol Derg	Out of the final scope of the review (i.e. on healthcare associated protozoa infection other than malaria)	Excluded after narrowing the scope of the review (focus on healthcare-associated malaria)
Wang L	Concurrent Infections of Giardia duodenalis, Enterocytozoon bieneusi, and Clostridium difficile in Children during a Cryptosporidiosis Outbreak in a Pediatric Hospital in China	2013	PLoS Neglected Tropical Diseases	Out of the final scope of the review (i.e. on healthcare associated protozoa infection other than malaria)	Excluded after narrowing the scope of the review (focus on healthcare-associated malaria)
Aygun G	Parasites in nosocomial diarrhoea: Are they underestimated?	2005	Journal of Hospital Infection	Out of the final scope of the review (i.e. on healthcare associated protozoa infection other than malaria)	Excluded after narrowing the scope of the review (focus on healthcare-associated malaria)
Alrifai SB.	Prevalence and etiology of nosocomial diarrhoea in children < 5 years in Tikrit teaching hospital.	2009	East Mediterr Health J	Out of the final scope of the review (i.e. on healthcare associated protozoa infection other than malaria)	Excluded after narrowing the scope of the review (focus on healthcare-associated malaria)
Sandokji A.M.	Infectious nosocomial diarrhea in the surgical wards: Role of parasites and microbes imply stool analysis	2009	Journal of Taibah University Medical Sciences	Out of the final scope of the review (i.e. on healthcare associated protozoa infection other than malaria)	Excluded after narrowing the scope of the review (focus on healthcare-associated malaria)
Ambrosioni J	Crytospridium Spp. diarrhea in HSCT-recipients	2010	Clinical Microbiology and Infection	Out of the final scope of the review (i.e. on healthcare associated protozoa infection other than malaria)	Excluded after narrowing the scope of the review (focus on healthcare-associated malaria)
Feng Y.	Extended outbreak of cryptosporidiosis in a pediatric hospital, China	2012	Emerg Infect Dis	Out of the final scope of the review (i.e. on healthcare associated protozoa infection other than malaria)	Excluded after narrowing the scope of the review (focus on healthcare-associated malaria)
Pandak, N.	A family outbreak of cryptosporidiosis: Probable nosocomial infection and person-to-person transmission	2006	Wiener Klinische Wochenschrift	Out of the final scope of the review (i.e. on healthcare associated protozoa infection other than malaria)	Excluded after narrowing the scope of the review (focus on healthcare-associated malaria)
Bruce, B. B	Risk of Cryptosporidium parvum transmission between hospital roommates	2000	Clinical Infectious Diseases	Out of the final scope of the review (i.e. on healthcare associated protozoa infection other than malaria)	Excluded after narrowing the scope of the review (focus on healthcare-associated malaria)

el-Sibaei M.M.	Nosocomial sources of cryptosporidial infection in newly admitted patients in Ain Shams University Pediatric Hospital	2003	Journal of the Egyptian Society of Parasitology	Out of the final scope of the review (i.e. on healthcare associated protozoa infection other than malaria)	Excluded after narrowing the scope of the review (focus on healthcare-associated malaria)
Bradbury R.S.	Prevalence of Acanthamoeba spp. in Tasmanian intensive care clinical specimens	2014	J Hosp Infect	Out of the final scope of the review (i.e. on healthcare associated protozoa infection other than malaria)	Excluded after narrowing the scope of the review (focus on healthcare-associated malaria)

Supplementary table 4. Quality assessment criteria and verdict

Criteria code	Criteria	Explanation of the criteria
C1	Was the hypothesis/aim/objective of the study clearly described?	0= No clear description of the hypothesis/aim/objective of the study, 1= A clear description of the hypothesis/aim/objective of the study
C2	Were the characteristics of the patient(s) included in the study clearly described?	0= No clear description of the patient characteristic was provided, 1 = general characteristics of the patients were clearly reported (e.g., age, gender, underlying conditions)
C3	Were the reason(s) for admission to the healtcare facility provided? Alternatively mention of the fact it is a healthcare worker (no admission)	0= No reasons provided, 1 = Reason(s) provided
C4	Were the investigations for source of infection attribution clearly described?	0= No clear description of the investigations was provided, 1 = A clear description of the investigations was provided
C5	Were arguments provided to exclude infection prior entering the healthcare facility and/or to categorise the case(s) as healthcare-associated?	0= No arguments were provided, 1 = Argument were provided
C6	Were different possible routes of transmission clearly discussed and evaluated?<br>Note: examples of routes are vectorial transmission, induced/iatrogenic transmission, from environmental contamination of the healthcare facility, transmission via contaminated food, and transmission via contaminated water.	0= No routes of transmission were discussed or evaluated, 1= Possible transmission routes were assessed and evaluated
C7	Were the (suspected) index case identified/search for and possible exposure to the (suspected) index case clearly described? For food and waterborne infections, was the source identified/search for and possible exposure to the source described?	0= No clear description of the exposure to the index case/source was provided, 1 = A clear description of the exposure to the index case/source was provided
C8	When multiple healthcare associated cases were described, was the possible route(s) of infection assessed for each case individually?	[ONLY WHEN MULTIPLE CASES ARE DESCRIBED] 0= The possible route(s) was/were not assessed for each case individually, 1= The possible route(s) was/were assessed for each case individually

Verdict criteria
1= Poor
2= Poor
3= Poor
4= Fair
5= Fair
6= Good
7= Good
8= Good

Supplementary table S6. Results on the quality assessment

Running number	First Author	Title	C1	C2	C3	C4	C5	C6	C7	C8	Total score	Verdict
1	Al-Hamdan, N. A.	Hospital-acquired malaria associated with dispensing diluted heparin solution	1	0	1	0	1	1	0	NA	4	Fair
2	Boccolini, D.	Non-imported malaria in Italy: paradigmatic approaches and public health implications following an unusual cluster of cases in 2017	1	1	1	1	1	0	1	NA	6	Good
3	Gonzalez, L.	Nosocomial Plasmodium falciparum infections confirmed by molecular typing in Medellín, Colombia	1	1	1	1	1	1	1	1	8	Good
4	Gruell, H.	On taking a diferent route: An unlikely case of malaria by nosocomial transmission	1	1	1	1	1	1	1	NA	7	Good
5	Jain, S.K.	Nosocomial malaria and saline flush	1	1	1	1	1	1	1	NA	7	Good
6	Lee, E.H.	Healthcare-associated transmission of plasmodium falciparum in New York City	1	1	1	1	1	1	1	NA	7	Good
7	Kim, J.Y.	A locally acquired falciparum malaria via nosocomial transmission in Korea	1	1	1	1	1	1	1	NA	7	Good
8	Kirchgatter, K.	Molecular typing of Plasmodium falciparum from Giemsa-stained blood smears confirms nosocomial malaria transmission	1	1	1	1	1	1	1	NA	7	Good
9	Moran, E.	Case of cryptic malaria	0	1	1	1	1	1	1	NA	6	Good
10	Piro, S.	Hospital-acquired malaria transmitted by contaminated gloves	1	1	1	1	1	1	1	1	8	Good
11	Winterberg, D.H.	A boy with nosocomial malaria tropica contracted in a Dutch hospital	1	1	1	1	1	1	1	NA	7	Good
12	Al-Saigul, A. M.	Nosocomial malaria from contamination of a multidose heparin container with blood	1	1	1	1	1	1	1	NA	7	Good
13	Alweis, R. L.	Serial Nosocomial Transmission of Plasmodium falciparum Malaria from Patient to Nurse to Patient	1	1	1	1	1	1	1	1	8	Good
14	Moro, M. L.	Patient-to-patient transmission of nosocomial malaria in Italy	1	1	1	1	1	1	1	NA	7	Good

15	Tarantola, A.	Occupational Plasmodium falciparum malaria following accidental blood exposure: A case, published reports and considerations for post-exposure prophylaxis	1	1	1	0	0	0	1	NA	4	Fair
16	Rotaeche Montalvo V	Rotaeche Montevaldo. Paludismo inducido en Espana	1	1	1	0	1	0	1	NA	5	Fair
17	Asgari, N.	A case of hospital acquired malaria in England	0	0	1	1	0	0	1	NA	3	Poor
18	European Centre for Disease Prevention and Control	Hospital-acquired malaria infections in the European Union	1	1	1	1	1	1	1	1	8	Good
19	Diguio, N.	Retour sur un cas de paludisme nosocomial	0	1	1	1	0	1	1	NA	5	Fair
20	National Public Health Organization, Greece	Annual epidemiological surveillance report, Malaria in Greece 2021	1	0	0	0	0	0	0	NA	1	Poor
21	National Public Health Organization, Greece	Annual epidemiological surveillance report, Malaria in Greece 2020	1	0	0	0	0	0	0	NA	1	Poor
22	National Public Health Organization, Greece	Epidemiological surveillance report, Malaria in Greece 2017	1	0	0	0	0	0	0	NA	1	Poor
23	Zoller, T.	Malaria transmission in non-endemic areas: case report, review of the literature and implications for public health management.	1	1	1	1	1	1	1	NA	7	Good
24	Fernández Martínez B	Situación del paludismo en España. Evolución del tipo de notificación a la Red Nacional de Vigilancia Epidemiológica y resumen de los resultados de la vigilancia de 2014 a 2017	1	0	0	0	0	0	0	NA	1	Poor

25	Centro Nacional de Epidemiología. Instituto de Salud Carlos III.	Resultados de la Vigilancia Epidemiológica de las enfermedades transmisibles. Informe anual. 2010	1	0	0	0	0	0	0	NA	1	Poor
26	Centro Nacional de Epidemiología. Instituto de Salud Carlos III.	Resultados de la Vigilancia Epidemiológica de las enfermedades transmisibles. Informe anual. 2011	1	0	0	0	0	0	0	NA	1	Poor
27	Verona, B.	Epidemiological investigation of a case of malaria in a non-endemic area, Campo de Gibraltar, Cadiz, Spain, January 2022	1	1	1	1	1	1	1	NA	7	Good
28	Coppée, R.	Nosocomial Malaria Transmissions Resolved by Genomic Analyses-A Retrospective Case Report Study in France: 2007-2021	1	1	1	0	1	1	1	1	7	Good
29	Centro Nacional de Epidemiología. Instituto de Salud Carlos III.	Resultados de la vigilancia epidemiológica de las enfermedades transmisibles. Informe anual. Años 2017-2018.	1	0	0	0	0	0	0	0	1	Poor
30	Centro Nacional de Epidemiología. Instituto de Salud Carlos III.	Informe epidemiológico sobre la situación de paludismo en España. Años 2019, 2020 y 2021	1	1	0	1	1	0	0	NA	4	Fair
31	Nacionalinė visuomenės sveikatos priežiūros laboratorija	NA; unpublished	NA	NA	NA	NA	NA	NA	NA	NA	NA	NA
32	Robert Koch Institute	Infektionsepidemiologisches Jahrbuch	1	0	1	0	0	0	0	NA	2	Poor

33	Centro Nacional de Epidemiología. Instituto de Salud Carlos III.	NA; unpublished	NA	NA	NA	NA	NA	NA	NA	NA	NA	NA
34	Istituto Superiore di Sanità	NA; unpublished	NA	NA	NA	NA	NA	NA	NA	NA	NA	NA
35	Istituto Superiore di Sanità	NA; unpublished	NA	NA	NA	NA	NA	NA	NA	NA	NA	NA
36	Istituto Superiore di Sanità	NA; unpublished	NA	NA	NA	NA	NA	NA	NA	NA	NA	NA
37	Istituto Superiore di Sanità	NA; unpublished	NA	NA	NA	NA	NA	NA	NA	NA	NA	NA

Supplement S1. List of the websites of regional and national public health authorities and of international health organisations that have been screened.

Africa Centres for Disease Control and Prevention

- https://africacdc.org/

Africa Health Organisation

- https://www.aho.org/

Argentina

- https://www.argentina.gob.ar/salud

Australia

- https://www.health.gov.au/
- https://www.phaa.net.au/

Austria

- https://www.ages.at/en/

Belgium

- https://www.health.belgium.be/fr
- https://www.sciensano.be/
- https://www.itg.be/

Bulgaria

- https://www.mh.government.bg/en/

Brazil

- https://www.gov.br/saude/en

Canada

- https://www.canada.ca/en/public-health.html

China

- http://en.nhc.gov.cn/
- https://en.chinacdc.cn/

Croatia

- https://www.hzjz.hr/en/

Cyprus

- https://www.moh.gov.cy/

Czechia

- https://www.mzcr.cz/en/the-ministry-of-health/

Egypt

- https://www.gah.gov.eg/indexenglish.php

Estonia

- https://www.terviseamet.ee/en
- https://tehik.ee/en

European Centre for Disease Prevention and Control

- https://www.ecdc.europa.eu/en

Finland

- https://thl.fi/en/web/thlfi-en

France

- https://sante.gouv.fr/
- https://www.sante.fr/
- https://www.cpias-ile-de-france.fr/

Germany

- https://www.rki.de/

Greece:

- https://www.eody.gov.gr/

Hungary

- https://egeszsegvonal.gov.hu/

Italy

- https://www.epicentro.iss.it/
- https://www.iss.it/

Iraq

- http://www.phd.iq/English/index.php

Ireland

- https://www.hse.ie/

Japan

- https://www.mhlw.go.jp/english/
- https://www.niph.go.jp/en/index/

Latvia

- https://www.zva.gov.lv/en

Lithuania

- https://sam.lrv.lt/en/
- https://nvsc.lrv.lt/en/

Malta

- https://health.gov.mt/

Montenegro

- https://www.gov.me/en/mzd

Netherlands

- https://www.rivm.nl/

New Zealand

- https://www.health.govt.nz/our-work/te-pou-hauora-tumatanui-public-health-agency

Norway

- https://www.fhi.no/en/

Poland

- https://www.urpl.gov.pl/en/office

Portugal

- https://www.insa.min-saude.pt
- https://www.dgs.pt/

Qatar

- https://www.moph.gov.qa/english/Pages/default.aspx

Romania

- https://ms.ro/en/

Russia

- https://minzdrav.gov.ru/en

Saudi Arabia

- https://www.moh.gov.sa/en/Pages/default.aspx
- https://www.my.gov.sa/wps/portal/snp/main

Serbia

- https://www.batut.org.rs/index.php?lang=2

Slovakia

- https://www.uvzsr.sk/web/uvzen

Slovenia

- https://www.jazmp.si/en/
- https://www.gov.si/en/state-authorities/ministries/ministry-of-health/

South Africa

- https://www.gov.za/about-sa/health
- https://www.phbsa.ac.za/

Spain

- https://www.isciii.es/Paginas/Inicio.aspx

Sweden

- https://www.folkhalsomyndigheten.se/

Switzerland

- https://public-health.ch/
- https://www.swisstph.ch/

Ukraine

- https://en.moz.gov.ua/about-the-ministry

United Kingdom

- https://www.gov.uk/government/organisations/public-health-england
- https://www.gov.uk/government/organisations/uk-health-security-agency
- https://www.gov.uk/government/organisations/office-for-health-improvement-and-disparities

United States

- https://www.cdc.gov/

World Health Organization

- https://www.who.int/
- https://www.who.int/europe/
- https://www.paho.org/en
- https://www.who.int/westernpacific
- https://www.who.int/southeastasia/about
- https://www.afro.who.int/
- https://www.emro.who.int/countries.html

Supplement S2. List of the countries contacted for the healthcare associated malaria data call.

In July 2023, we contacted the public health institutes in

Albania, Austria, Belgium, Bosnia and Herzegovina, Bulgaria, Croatia, Cyprus, Czech Republic, Denmark, Estonia, Finland, France, Germany, Greece, Hungary, Iceland, Ireland, Italy, Kosovo*, Latvia, Liechtenstein, Lithuania, Luxembourg, Malta, Netherlands, Montenegro, North Macedonia, Norway, Poland, Portugal, Romania, Serbia, Slovakia, Slovenia, Spain, Sweden, Türkiye.

* This designation is without prejudice to positions on status, and is in line with UNSCR 1244/1999 and the ICJ Opinion on the Kosovo Declaration of Independence
